# Improving Stockline Detection of Radar Sensor Array Systems in Blast Furnaces Using a Novel Encoder–Decoder Architecture

**DOI:** 10.3390/s19163470

**Published:** 2019-08-08

**Authors:** Xiaopeng Liu, Yan Liu, Meng Zhang, Xianzhong Chen, Jiangyun Li

**Affiliations:** 1 School of Automation & Electrical Engineering, University of Science and Technology Beijing, Beijing 100083, China; 2 Key Laboratory of Knowledge Automation for Industrial Processes, Ministry of Education, Beijing 100083, China; 3 Instrument Science & Technology, Beijing 100083, China

**Keywords:** sensor array system, blast furnace, stockline detection, radar signal processing, LSTM

## Abstract

The stockline, which describes the measured depth of the blast furnace (BF) burden surface with time, is significant to the operator executing an optimized charging operation. For the harsh BF environment, noise interferences and aberrant measurements are the main challenges of stockline detection. In this paper, a novel encoder–decoder architecture that consists of a convolution neural network (CNN) and a long short-term memory (LSTM) network is proposed, which suppresses the noise interferences, classifies the distorted signals, and regresses the stockline in a learning way. By leveraging the LSTM, we are able to model the longer historical measurements for robust stockline tracking. Compared to traditional hand-crafted denoising processing, the time and efforts could be greatly saved. Experiments are conducted on an actual eight-radar array system in a blast furnace, and the effectiveness of the proposed method is demonstrated on the real recorded data.

## 1. Introduction

Blast furnaces (BFs) are the key reactors of iron and steel smelting, which consumes about 70% of the energy (i.e., coal, electricity, fuel oil, and natural gas) in the steel-making process [1,2]. In iron-making, solid raw materials, e.g., iron ore, coke, limestone, are violently burned and consumed from time to time, and the charging operation needs to be executed by accurately estimating the current depth of the burden surface. Burden surface monitoring is crucial to ensuring high quality steel production as it affects the optimization of charging operations and the utilization ratio of heat and chemical energy [3].

**Figure 1:**
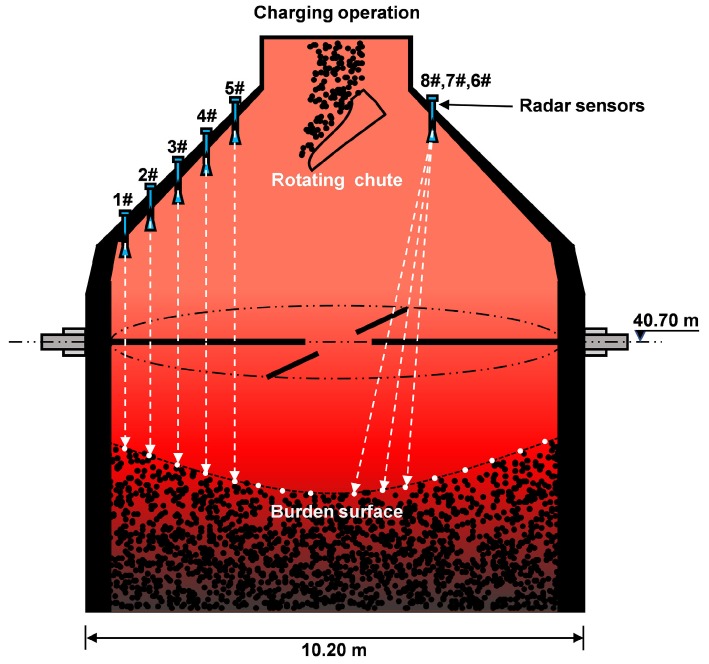
Prototype of the employed eight-radar array system. The frequency-modulated continuous wave (FMCW) radars work in the band of 24 GHz∼26 GHz, with a bandwidth of 1.64 GHz. The radar sensors are placed by scientifically considering the practical industrial field.

The measurement of the BF burden surface is a longstanding and challenging task because of the harsh in-furnace environment, which is lightless, high-pressure, high-dust, high-humidity, and extremely high in temperature [4]. With the advantage of its contact-free nature, high precision, and high penetrability, the frequency-modulated continuous wave (FMCW) radar has become widely popular with its pointwise measuring method to locate the burden level in real time [5]. As shown in Figure 1, the employed eight-radar array system is introduced, where the radar sensors are placed scientifically in consideration of the practical industrial field. The burden surface is bilaterally symmetrical because of the uniform rotation of the charging chute, so only half of the surface needs to be measured. The trajectory of the measuring points taken by the radar over time is termed the stockline and reflects the depth of the burden surface at every timestamp. Improving the precision of stockline detection, the operator can optimize the burden distribution in a way that is conducive to a stable iron-making process.

Noise interferences are crucial issues in the BF radar system. The radar signals usually suffer heavy low-frequency noises due to strong electromagnetic scattering. At times, the shadows of the rotating chute and falling materials, the influence of natural deviations, instrument errors, fraudulent behaviors, and other unexpected interferences result in the appearance of distorted signals and make stockline detection more challenging.

In the study of BFs, a variety of algorithms of noisy data processing have been developed so far, covering principal component analysis [6], support vector machines (SVM) [7,8], neural network models [9], extreme learning machines [10], etc. It is acknowledged by remarkable previous works that the noises occurring within the BF reactor are still an extremely complex issue. The existing works of stockline detection are reviewed [5,11,12,13,14,15]. Generally, peak searching (PS) is used to extract the stockline, which corresponds to the maximum amplitude component in the signal spectrum [12]. In the case of actual BF environments, the collected signals are usually corrupted with noises that bury the target features under fraudulent peaks and bring outliers to the stockline. A simple approach to eliminate the noise influence is threshold clipping, of which the adaptive threshold method is an example [13], where the noises outside the thresholds would be ignored rudely. Effective filtering methods are being developed to exclude the empirical interval of noise distribution, e.g., the infinite impulse response (IIR) filter used in [14] and the windowed finite impulse response (FIR) filter used in [16]. Ongoing efforts are being made to improve noise robustness by taking historical observations into consideration, e.g., Kalman tracking methods [17,18]. Several stockline smoothing methods are presented to reduce noise fluctuation, e.g., mean shift and spectrum average [13]. The CLEAN and clustering algorithms, as reported in [5], can omit the falsely noisy targets on the burden surface but are unsuitable for the task of continuous detection as such. Traditional noise abatement requires domain expertise to construct the feature selector, involving, for example, threshold selection or noise filtering, to the extent that it limits further performance improvement. Another bottleneck is the limited stockline tracking capability.

Recently, deep neural networks have made considerable progress on diverse kinds of data processing, such as image, video, speech, and text [19]. The convolutional neural network (CNN) is widely believed to have a powerful learning ability for feature selection and extraction. Besides, with the advantage of long-range memory, the long short-term memory (LSTM) network is broadly used in time-series data modeling. An increasing number of hybrid architectures that combine the CNN and LSTM as an encoder–decoder pair have achieved great success in promoting long-term information learning, such as the image caption [20], speech signal processing [21,22], sensory signal estimation [23], etc.

In this paper, we propose a novel encoder–decoder architecture for effective stockline detection. The encoder is a one-dimensional convolutional neural network (1D-CNN), and the decoder is a cascade multi-layer LSTM network. Between the encoder and decoder, a binary classifier is constructed to mitigate the negative impacts of distorted signals. The hybrid architecture has an excellent anti-noise learning ability and a long-range stockline tracking ability. Our contributions can be summarized as follows:

- To present a novel encoder–decoder architecture to improve stockline detection, which learns desired features from noisy data adaptively. We save time and effort compared to traditional hand-crafted denoising processing.
- To present an effective stockline tracking strategy by leveraging the LSTM network to model longer range historical signals. A large tracking capability brings better robustness of noise randomness.
- The experiments are validated on actual industrial BF data. In particular, the experiments are carried out on an intact multi-radar scenario rather than a single radar scenario.

The rest of the paper is organized as follows. In the second section, the issues of stockline detection and the necessity of the encoder–decoder architecture are described. The proposed algorithm and the loss function are explained in Section 3. We conduct the experiments on actual BF data collected from the eight-radar array system in Section 4. A conclusion is provided in Section 5 to summarize this work.

## 2. Issue Description and Necessity Of Encoder-Decoder Architecture

**Figure 2:**
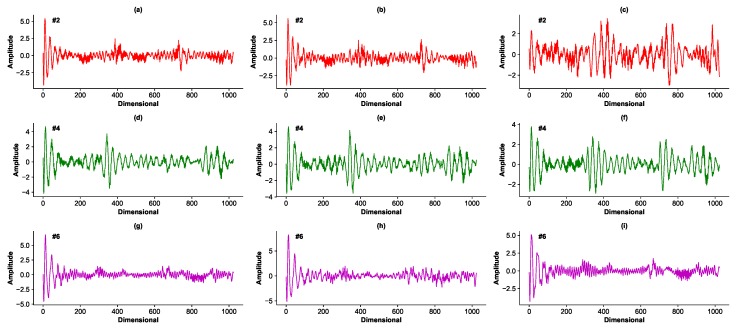
Examples of the input signals. Each signal is 1024-dimensional. (**a**–**c**) are examples from radar #2; (**d**–**f**) are examples from radar #4; (**g**–**i**) are examples from radar #6.

**Figure 3:**
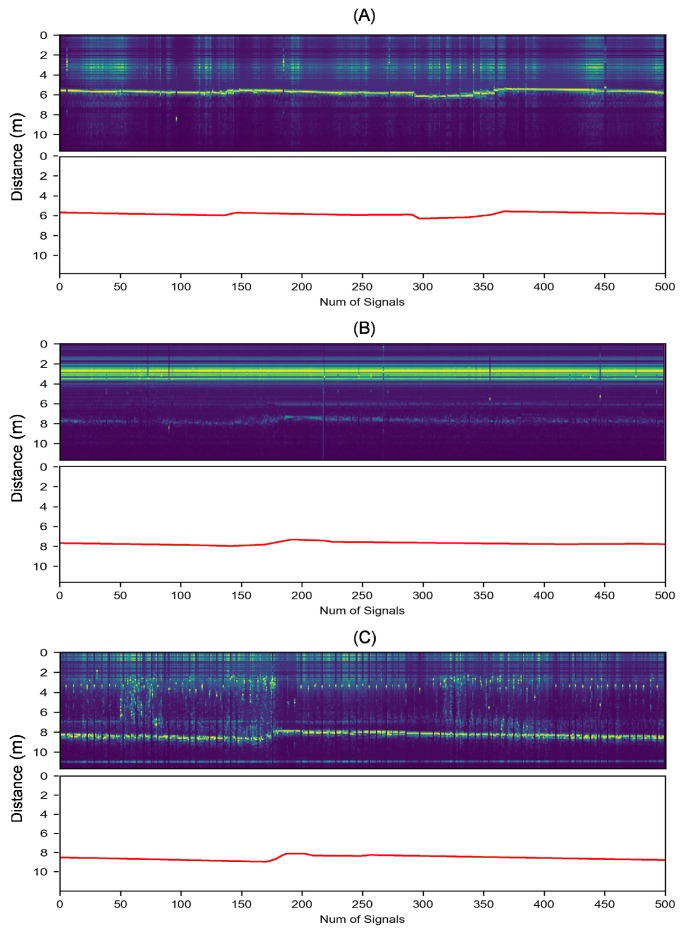
The time–frequency spectrum and the expectation stockline of different radar signals. (**A**–**C**) are from radar #2, #4, and #6, respectively. The horizontal axis represents the number of time series signals, and the vertical axis represents the distance between the radar sensor and the measured point.

The signals are collected individually and sequentially among different radars. They are 1024-dimensional vectors quantified by the 10-bit analog–digital converter. There are some examples of the input signals shown in Figure 2. Stocklines are fluctuant curves that reflect the changing depth of the burden surface, as shown in the radar temporal-frequency spectrum in Figure 3 where the vertical coordinates have been converted to the measuring distance using

(1) $$d=\frac{c}{2B}\cdot f,$$

where *d* is the distance in m, $c=3\times 10^{8}$ m/s is the velocity of electromagnetic waves, and B=1.64 GHz represents the radar bandwidth. *f* is the beat frequency in Hz. More introductions about the FMCW radar can be found in [24].

Several typical noisy conditions are depicted in Figure 3. Figure 3A shows a condition where the stockline is able to be detected. On the contrary, the stockline in Figure 3B appears to be buried under the strong low-frequency noises (the luminous yellow band above the stockline). In Figure 3C, the stockline is so discontinuous that numerous absent measurements occurred due to the interruption of distorted signals. In general, the collected data close to the furnace center suffer heavier noisy impacts compared to those close to the furnace wall.

As declared in [2,7], the unknown statistic properties of the actual BF noises are always a dilemma for noise abatement. Different from traditional methods, which perform data filtering processing that is heuristic, repetitive, and knowledge-based for a multi-radar system, the proposed method circumvents the noise interferences in a learning fashion, significantly reducing the time and effort of hand-engineered data filtering. The front CNN is used to extract representative features from the raw signals; the middle classifier is used to separate the fraudulent distorted signals; the trailing LSTM is utilized to capture the dependencies of time series measurements and decode the feature. Such an effective encoder–decoder (CNN–LSTM) backbone architecture tailored to stockline detection is presented.

## 3. Methodology

### 3.1. Architecture

**Figure 4:**
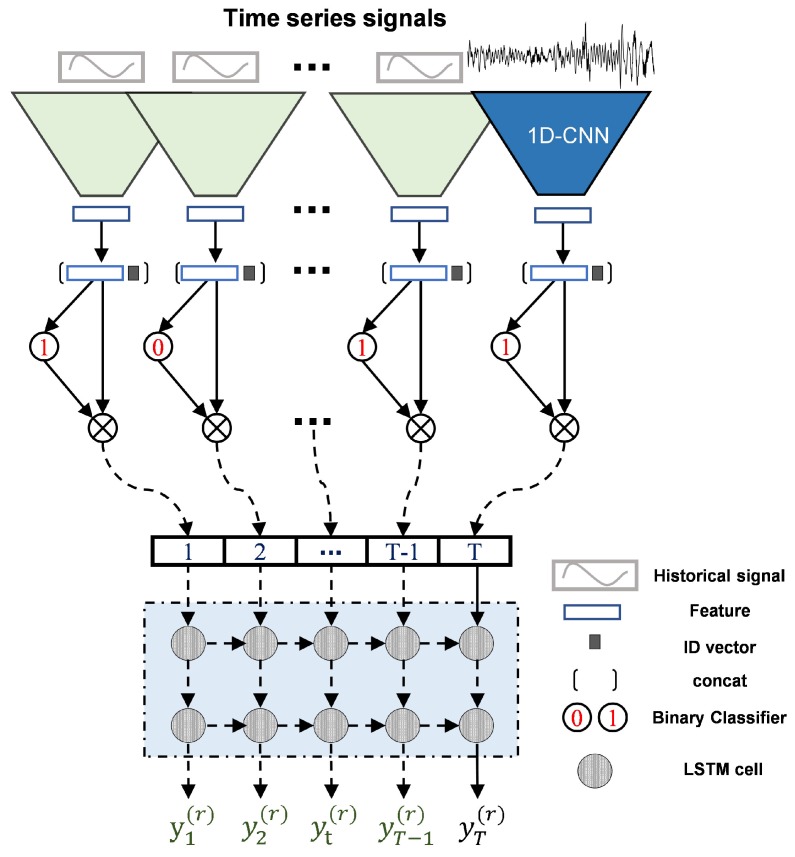
The proposed encoder–decoder architecture unrolled in time. LSTM: long short-term memory; 1D-CNN: one-dimensional convolutional neural network.

The proposed architecture is shown in Figure 4. It consists of four operations, including CNN feature extraction, radar identification (ID) embedding, distorted signal separation, and temporal feature decoding.

**Figure 5:**
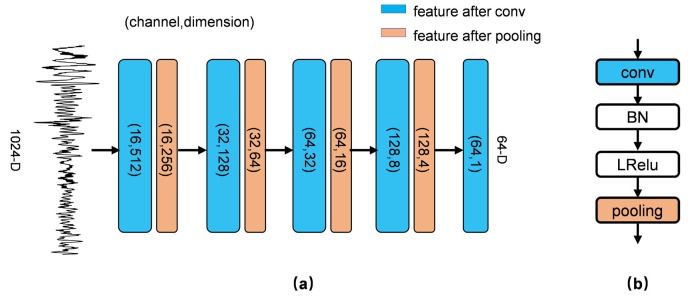
(**a**) The structure of the 1D-CNN encoder. It is composed of 5 convolutional layers and 4 pooling layers. Eight-length convolutional kernels are used at the first four layers, while 4-length kernels are used at the last layer. (**b**) We perform batch normalization (BN) and a leaky rectified linear unit (LRelu) function after each convolutional layer.

**CNN Encoder**. A five-layer CNN is constructed as the encoder. It receives the raw signal (1024-D) as input. The structure is shown in Figure 5a.

Mathematically, given the input of the convolutional layer, $X\in R^{C\times D}$, which is *C*-channel and *D*-dimensional. Let $W\in R^{K\times C\times L}$ be the weight, where *K* is the number of convolutional kernels and *L* is the kernel length. The 1D-convolutional calculation can be formulated as

(2) $$W*X={\left(\sum_{c\in C}\sum_{l\in L}W_{k,c,l}X_{c,i:i+L}\right)}_{\overset{i=1,1+s,\cdots ,1+\left\lceil \frac{D-L}{s}\right\rceil s}{k=1,2,\cdots ,K}},$$

where the symbol ∗ stands for the convolutional operation, *s* is the sliding stride, and $\left\lceil \cdot \right\rceil$ is the ceiling function.

We apply batch normalization (BN) and nonlinearity after each convolutional layer, as shown in Figure 5b. BN is widely used in the neural networks and can speed up convergence and slightly improve performance [25]. Assuming *x* is the input of BN, we have

(3) $${BN}_{\gamma ,\beta }\left(x\right)=\gamma \frac{x-E[x]}{\sqrt{Var[x]+\epsilon }}+\beta ,$$

where γ is a scaling factor and β is a shifting factor. It is noted that β also acts as a bias term to the convolutional layer. E[·] is the mean, and Var[·] is the variance. ϵ is a small positive constant to prevent division by zero, e.g., $\epsilon =10^{-5}$.

The leaky rectified linear unit (LRelu) is adopted as nonlinearity [26]. It is

(4) $$f\left(x\right)=\left\{\begin{array}{cc} x & \mathrm{if}\;x\geq 0,\\ \alpha x & \mathrm{otherwise}, \end{array}\right.$$

where f(·) represents the LRelu function and α is a constant, e.g., α=0.2.

Max-pooling layers with a stride of 2 are used in the encoder. After encoding by the CNN, the *m*-dimensional feature is extracted. *m* is a optional hyperparameter, and m=64 is used.

**ID Embedding**. The characteristics of noisy signal data are different from radar to radar. For multi-radar data, we embed the radar ID information into feature *F* to slightly improve performance. The radar ID is encoded using one-hot coding. Formally, given *R* radars, the corresponding ID is denoted by $I^{\left(r\right)}=\left(I_{1}^{\left(r\right)},\cdots ,I_{R}^{\left(r\right)}\right)$, r=1,2,⋯,R, where $I_{i}^{\left(r\right)}$ is the *i*-th component of $I^{\left(r\right)}$. For the *r*-th radar, we have

(5) $$I_{i}^{\left(r\right)}=\left\{\begin{array}{cc} 1 & \mathrm{if}\;i=r,\\ 0 & \mathrm{otherwise}. \end{array}\right.$$

$F^{\left(r\right)}$ and $I^{\left(r\right)}$ are concatenated together in dimensionality, the superscript denotes the signal derived from the *r*-th radar.

(6) $${\tilde{F}}^{\left(r\right)}=\left[F^{\left(r\right)};I^{\left(r\right)}\right]\in R^{m+R}.$$

**Distorted Signal Separation**. We construct a binary classifier using a fully connected layer to classify the normal signals and distorted signals before feeding them into the decoder. The fully connected layer maps the feature into a *V*-class decision space (V=2 for the 2-class task). Let $W\in R^{V\times \left|\tilde{F}\right|}$ be the weight, with $b=\left(b_{1},\cdots ,b_{V}\right)\in R^{V}$ as the bias. We have

(7) $$p=\mathrm{Softmax}{\left(\sum_{j\in \left|\tilde{F}\right|}W_{i,j}\cdot {{\tilde{F}}^{\left(r\right)}}_{j}+b_{i}\right)}_{i=1,\cdots ,V},$$

where ${{\tilde{F}}^{\left(r\right)}}_{j}$ is *j*-th element of ${\tilde{F}}^{\left(r\right)}$, $\left|\tilde{F}\right|$ is the dimensionality of ${\tilde{F}}^{\left(r\right)}$, and $p=(p_{1},\cdots ,p_{V})$ is the category predicting the probability by the softmax function. For the binary classification, we have the expectation $p^{*}=\left(1,0\right)$ for the normal signals and $p^{*}=\left(0,1\right)$ for distorted signals. The distorted signals carry confusing information and do not make any meaningful contributions to stockline estimation. We mask them by zero to reduce their negative impacts for the next calculation step.

**Figure 6:**
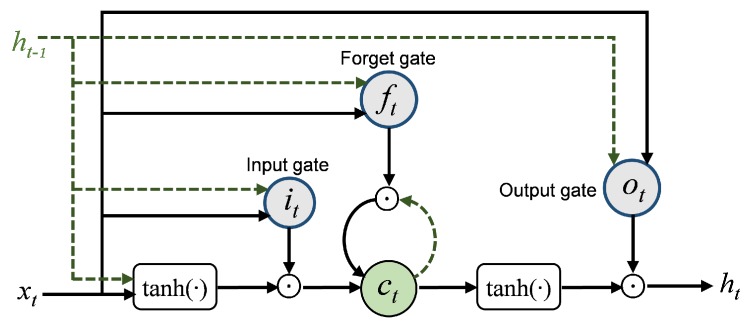
Diagram of an LSTM cell with its inner memory mechanism, including the input gate $i_{t}$, forget gate $f_{t}$, and output gate $o_{t}$. The dashed line represents the information from the last time step.

**LSTM Decoder**. LSTM is an improved variant of recurrent neural networks (RNNs) [27,28]. Define *T* be the tracking length of the stockline, including T−1 historical signals and one current signal. Let the subscript *t* be the index of the *T* sequential signals that are fed into the LSTM, so t=1,2,⋯,T. LSTM makes use of an effective gate mechanism to control the context information flow, which is comprised of the input gate $i_{t}$, the forget gate $f_{t}$, and the output gate $o_{t}$. Its inner gate mechanism is shown in Figure 6. The cell state $c_{t}$ stores the information of each time step, the input gate determines whether to add new information to $c_{t}$, the forget gate selectively forgets the uninteresting previous information involved in $c_{t}$, and they control the update of the cell state $c_{t}$. The output gate controls the emission from $c_{t}$ to the hidden state $h_{t}$. $h_{t}$ is mapped to the decoder output $y_{t}=U_{t}h_{t}+b_{t}$, where $U_{t}$ is the weight matrix and $b_{t}$ is the bias.

The output $y_{T}$ of LSTM would correspond to one point of the stockline. Equally, $y_{T}=\mathrm{LSTM}\left(x_{T}|x_{1},x_{2},\cdots ,x_{T-1}\right)$.

### 3.2. Loss Function

**Classification loss**. The classifier is trained by minimizing the cross-entropy loss. It can be formulated as

(8) $$\ell_{cls}\left(\theta \right)=-\frac{1}{T}\sum_{t\in T}\sum_{i\in V}p_{t,i}^{*}\log\left(p_{t,i}\right).$$

**Regression loss**. The output values ${\left\{y_{t}\right\}}_{t=1}^{T}$ are regressed to the target values ${\left\{y_{t}^{*}\right\}}_{t=1}^{T}$ by minimizing their square errors, and the form of regression loss is

(9) $$\ell_{reg}\left(\theta \right)={∥y_{T}-y_{T}^{*}∥}_{2}^{2}+\frac{\rho }{T-1}\sum_{t\in T-1}{∥y_{t}-y_{t}^{*}∥}_{2}^{2},$$

where ρ is the discount factor, ρ=0.5 is used.

**Joint loss**. It is a joint training task of classification and regression. The general way of constructing a joint loss is using a linear weighted sum of each subtask loss. The total loss can be written as

(10) $$\ell_{total}\left(\theta \right)=\lambda_{1}\ell_{reg}\left(\theta \right)+\lambda_{2}\ell_{cls}\left(\theta \right)+\lambda_{3}\ell_{norm}\left(\theta \right),$$

where $\lambda_{1},\lambda_{2},\lambda_{3}$ are the weighted factors of each subtask. $\ell_{norm}\left(\theta \right)=∥\theta ∥$ is the L2 norm loss.

When it simply presets $\lambda_{1}:\lambda_{2}$ to 1:1 in our experiments, the classification loss tends to steer the training process and damage the regression performance. Thus, we use the strategy of homoscedastic uncertainty to search for the suitable weight factors to balance their performance [29,30]. The joint loss is rewritten as

(11) $$\ell_{total}\left(\theta ,\sigma_{1},\sigma_{2}\right)=\frac{1}{2\sigma_{1}^{2}}\ell_{reg}\left(\theta \right)+\frac{1}{\sigma_{2}^{2}}\ell_{cls}\left(\theta \right)+\lambda_{3}\ell_{norm}\left(\theta \right)+\log\sigma_{1}\sigma_{2},$$

where $\sigma_{1}$ and $\sigma_{2}$ are two learnable parameters that stand for the observed variances of the subtasks. The bound term $\log\sigma_{1}\sigma_{2}$ discourages the variances from increasing too much. Assuming $\lambda_{1}=\frac{1}{2\sigma_{1}^{2}}$, $\lambda_{2}=\frac{1}{2\sigma_{2}^{2}}$, $\lambda_{3}=5\times 10^{4}$. On implementation, the network is trained to predict the log variance because it is more numerically stable; for instance, letting $s_{1}=\log\sigma_{1}^{2}$, we use $\sqrt{e^{s_{1}}}$ to replace $\sigma_{1}$ in (11), and so for $\sigma_{2}$.

## 4. Experiment

In this section, the experiments are carried out and the experimental setups are provided in detail. The experimental results consist of four parts: performance, scientificity of the architecture, the effect of the tracking strategy, and the running time.

### 4.1. Experiment Setup

**Dataset**. There are 504,488 signals collected from the industrial BF eight-radar array system as posed in Table 1. The data is divided into three parts, including the training set, validation set, and testing set; they are 3:1:1, respectively. The training set is used to train the model, the validation set is used to validate the performance and tune the hyperparameters, and the testing set is used to test its performance. The results are from the testing set as default, unless otherwise stated. The radar signals are normalized between [0,1] in order to eliminate the effect of the amplitude.

**Hyperparameters**. The encoder with 5 convolutional layers, the decoder with 3 hidden layers, 72 time steps (i.e., T=72), and 128 hidden nodes are adopted. The convolutional layers and fully connected layers are initialized with zero bias and a Gaussian weight filled with (−0.1,0.1). The LSTM cells are zero-initialized. We use the Adam solver [31], which is an improved stochastic gradient descent algorithm, to optimize the model. A dynamic learning rate is used,. It starts with 0.001 and decays by 0.98 per every 100 iterations. The model is trained for 20 epochs with a batch size of 120. We perform the dropout with a 50% dropout rate to the LSTM as regularization [28,32]. When the training is completed, the adaptive parameters $s_{1}$, $s_{2}$ are leveled off to −4.09, −0.34, respectively; in other words, $\lambda_{1}:\lambda_{2}=29.87:1.40$.

**Evaluation**. We use mean absolute error (MAE) and root mean square error (RMSE) to evaluate the detection performance. Formally, MAE is defined as

(12) $$\mathrm{MAE}\left(y,y^{*}\right)=\frac{1}{N}\sum_{i\in N}\left|y_{i}-y_{i}^{*}\right|\;.$$

The RMSE is defined as

(13) $$\mathrm{RMSE}\left(y,y^{*}\right)=\sqrt{\frac{1}{N}\sum_{i\in N}{∥y_{i}-y_{i}^{*}∥}_{2}^{2}}\;.$$

We use accuracy, precision, recall, and F1 score to evaluate the classification performance. Accuracy indicates the proportion of correctly classified samples among all samples. Precision indicates the proportion of true positive samples among true positive samples (TPs) and false positive samples (FPs), that is,

(14) $$\mathrm{precision}=\frac{\mathrm{TP}}{\mathrm{TP}\;+\;\mathrm{FP}}.$$

Recall indicates the proportion of true positive samples among true positive samples and false negative samples (FNs).

(15) $$\mathrm{recall}=\frac{\mathrm{TP}}{\mathrm{TP}\;+\;\mathrm{FN}}$$

The F1 score is the harmonic mean of precision and recall.

(16) $$F_{1}=\frac{2\cdot \mathrm{precision}\cdot \mathrm{recall}}{\mathrm{precision}\;+\;\mathrm{recall}}$$

### 4.2. Results

Different experiments were conducted as shown below. The proposed method was implemented on the Tensorflow framework [33].

**Performance**. The traditional peak searching method with pass-band FIR filters and Kalman filters were implemented for comparison. The settings of the FIR filters are shown in Table 2, and the Kalman algorithm is introduced in [34]. Respectively, the single CNN model and single LSTM model were constructed for another baseline comparison and their structures were identical to the corresponding part of the hybrid model.

**Figure 7:**
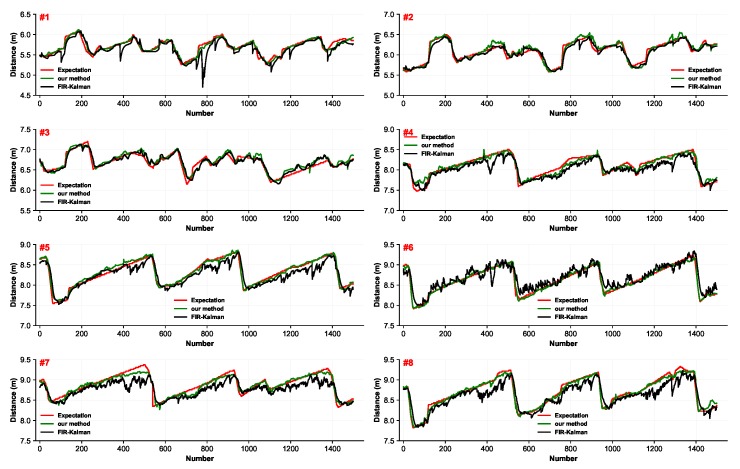
The estimation stocklines of the eight-radar array system measured in the same period.

The estimation stocklines are displayed in Figure 7, including the proposed method and FIR-Kalman methods. The regression curves of the proposed method exhibited less fluctuation around the expectation curves. In contrast, the filter-based approaches tended to be influenced by interferences.

As shown in Table 3 and Table 4, with an MAE of 0.0432 and a RMSE of 0.0581, the presented architecture is capable of learning features and suppressing noise interferences of noisy data. It could be observed that the proposed method significantly outperforms traditional experience-dependent denoising methods. From the table, the traditional method without denoising processing is extremely sensitive to heavy noisy impacts and achieves a poor performance. In contrast, the proposed method shows itself to be more efficient and robust.

The classifier performance is shown in Table 5. With scores of 95.90%, 96.00%, 99.48%, and 97.68% for the accuracy, precision, recall, and F1 score indicators, respectively, it achieves a decent classification result that can classify the distorted signals well.

**Scientificity of architecture**. A series of experiments were carried out to verify the scientificity of such an architecture. First is the validity of such a CNN–LSTM mixed architecture. Two simpler baseline architectures are provided, i.e., a single CNN architecture and a single LSTM architecture, as shown earlier in Table 3 and Table 4. The detached architectures both show worse performances compared to the proposed mixed architecture due to losing the advantages of each. For the simple CNN architecture, it shows a weak performance and is even worse than traditional denoising methods. For the simple LSTM architecture, we observe that LSTM gets into trouble learning the feature from noisy signals without the help of the CNN encoder.

**Figure 8:**
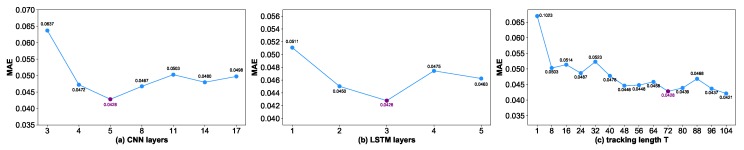
Performance on the validation set. (**a**) Performance of selecting different encoder layers. (**b**) Performance of selecting different decoder layers. (**c**) Performance of selecting different tracking lengths *T*.

Secondly, we conduct experiments to examine the complexity of the encoder and decoder. The impacts of picking a different number of CNN layers and a different number of LSTM layers are shown in Figure 8a,b, respectively. Based on the experiments, we choose a 5-layer CNN and a 3-layer LSTM as the encoder and decoder for optimal performance. The experimental results also indicate that a deep architecture is not always necessary.

Thirdly, the stockline tracking length *T* is examined. The experiments are shown in Figure 8c; as *T* increases, errors decrease in a general trend. Due to the limited memory of the LSTM network, it is found that when the tracking length T>72, it brings a marginal improvement of performance but conspicuously increases computational time, so a proper T=72 is used.

**Figure 9:**
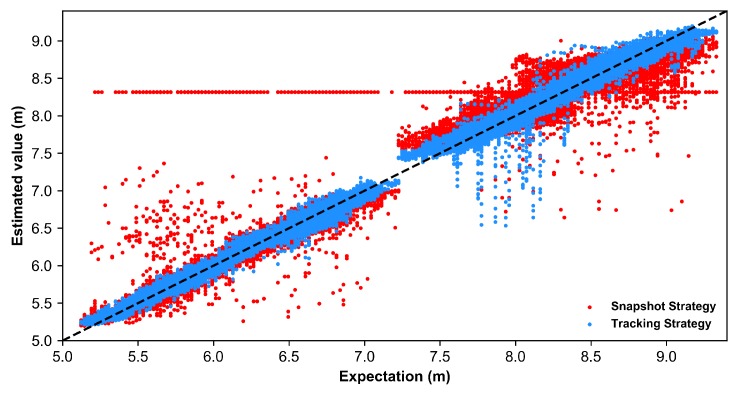
The distribution of the estimated values by the snapshot-based model and tracking-based model. As can be seen in the figure, a group of fixed false estimated points (red) occurred at ∼8.3 m, perhaps caused by a fixed noisy target within the blast furnace (BF).

**Effect of tracking strategy**. To further validate the effect of the stockline tracking ability provided by the LSTM, a snapshot-based model as designed by setting the time step T=1, implying that the network has no visible historical signal. We compare its performance with the tracking-based model (i.e., T=72) with fair training settings. The results are shown in Figure 9, where the black dotted line stands for the expectation, and the one whose distribution is closer to the black line shows a better performance. The snapshot-based tracking strategy, with a MAE of 0.1019 and a RMSE of 0.1854, is more sensitive to the interruptive noises and is even worse than the FIR-Kalman approach. As shown earlier in Figure 8c, a longer tracking capability has a positive effect on performance. The proposed method takes a longer range of previous stockline into consideration; however, the Kalman tracking approach only makes use of one previous moment. An effective tracking strategy brings a better tolerance to noisy randomness and disturbances.

**Running time**. In addition, with an average computational time of 0.522 ms, the proposed architecture is lightweight and fully meets the real-time requirements of the industrial process.

## 5. Conclusions

In this paper, we have successfully developed a hybrid CNN–LSTM architecture for the challenging stockline detection problem. Its effectiveness has been demonstrated by experiments on BF eight-radar array data. The success of the proposed method is attributable to three reasons: its effective learning ability, its ability to classify distorted signals, and its excellent stockline tracking ability.

In the industrial process, most of the monitoring data or variables have close time-series dependencies. The general contribution of this work is that we improve the long-range history learning by leveraging the novel LSTM network. It is a very promising direction to achieve more accurate and robust industrial control, if we can make the most of the context relationship of successive measurements.

In future work, we will dedicate our efforts to the image reconstruction of BF burden surfaces. The inner transparency of the neural network methodology will be investigated, which will allow us to have an in-depth qualitative or quantitative analysis of noise influences.

## Figures and Tables

**Table 1:** The number of normal signals and distorted signals.

Radar ID	Normal	Distorted	Total
1#	61,904	1157	63,061
2#	61,267	1794	63,061
3#	61,715	1346	63,061
4#	50,490	12,571	63,061
5#	53,599	9462	63,061
6#	52,150	10,911	63,061
7#	52,719	10,342	63,061
8#	53,839	9222	63,061

**Table 2:** Configuration of the FIR filters with the Blackman–Harris windows.

Radar ID	1#	2#	3#	4#	5#	6#	7#	8#
Window Length	64	96	264	136	136	128	184	104

**Table 3:** Comparison of the proposed method with different methods on the testing set (mean absolute error, MAE). PS: peak searching.

Radar ID	PS (w/o Denoising)	FIR-PS	FIR-Kalman-PS	CNN	LSTM	CNN-LSTM (Ours)
1#	0.2575	0.0825	0.0733	0.1153	0.1675	**0.0320**
2#	0.7230	0.0621	0.0502	0.1751	0.1952	**0.0434**
3#	0.5422	0.0898	0.0583	0.2403	0.1938	**0.0520**
4#	5.1153	0.1330	0.0892	0.1986	0.2304	**0.0645**
5#	5.1069	0.1030	0.0778	0.1434	1.1906	**0.0418**
6#	1.4011	0.1629	0.0938	0.1741	0.2822	**0.0318**
7#	5.8221	0.1433	0.1032	0.2010	0.2044	**0.0414**
8#	0.9030	0.1301	0.1132	0.1601	0.2967	**0.0385**
Average	2.8097	0.1133	0.0824	0.1760	0.3451	**0.0432**

**Table 4:** Comparison of the proposed method with different methods on testing set (root mean square error, RMSE).

Radar ID	PS (w/o Denoising)	FIR-PS	FIR-Kalman-PS	CNN	LSTM	CNN-LSTM (Ours)
1#	0.8002	0.2776	0.1130	0.2770	0.2082	**0.0423**
2#	3.2089	0.1321	0.0640	0.2900	0.2384	**0.0564**
3#	1.3763	0.1445	0.0738	0.2997	0.2458	**0.0675**
4#	5.1337	0.2300	0.1145	0.2666	0.2853	**0.0869**
5#	5.2721	0.2191	0.1033	0.2264	1.5000	**0.0588**
6#	2.7862	0.3662	0.1235	0.2645	0.3404	**0.0427**
7#	5.8500	0.2282	0.1317	0.2907	0.2509	**0.0559**
8#	2.0667	0.2994	0.1438	0.2547	0.3605	**0.0540**
Average	3.5598	0.2371	0.1084	0.2712	0.4287	**0.0581**

**Table 5:** Classification performance on testing set.

Radar ID	Accuracy	Precision	Recall	F1 Score
1#	98.33%	98.36%	99.98%	99.16%
2#	98.84%	98.97%	99.86%	99.42%
3#	97.97%	98.14%	99.81%	98.97%
4#	94.48%	96.08%	97.67%	96.87%
5#	89.07%	88.05%	99.99%	93.64%
6#	95.83%	95.41%	99.81%	97.56%
7#	95.62%	96.14%	98.85%	97.48%
8#	97.08%	96.88%	99.90%	98.37%
Average	95.90%	96.00%	99.48%	97.68%
